# Discovery of a monophagous true predator, a specialist termite-eating spider (Araneae: Ammoxenidae)

**DOI:** 10.1038/srep14013

**Published:** 2015-09-11

**Authors:** Lenka Petráková, Eva Líznarová, Stano Pekár, Charles R. Haddad, Lenka Sentenská, William O. C. Symondson

**Affiliations:** 1 Department of Botany and Zoology, Faculty of Science, Masaryk University, Kotlářská 2, 611 37 Brno, Czech Republic; 2 Department of Zoology & Entomology, University of the Free State, P.O. Box 339, Bloemfontein 9300, South Africa; 3 Cardiff School of Biosciences, Cardiff University, Sir Martin Evans Building, Museum Avenue, Cardiff CF10 3AX, United Kingdom

## Abstract

True predators are characterised by capturing a number of prey items during their lifetime and by being generalists. Some true predators are facultative specialists, but very few species are stenophagous specialists that catch only a few closely related prey types. A monophagous true predator that would exploit a single prey species has not been discovered yet. Representatives of the spider family Ammoxenidae have been reported to have evolved to only catch termites. Here we tested the hypothesis that *Ammoxenus amphalodes* is a monophagous termite-eater capturing only *Hodotermes mossambicus*. We studied the trophic niche of *A. amphalodes* by means of molecular analysis of the gut contents using Next Generation Sequencing. We investigated their willingness to accept alternative prey and observed their specific predatory behaviour and prey capture efficiency. We found all of the 1.4 million sequences were *H. mossambicus*. In the laboratory *A. amphalodes* did not accept any other prey, including other termite species. The spiders attacked the lateral side of the thorax of termites and immobilised them within 1 min. The paralysis efficiency was independent of predator:prey size ratio. The results strongly indicate that *A. amphalodes* is a monophagous prey specialist, specifically adapted to feed on *H. mossambicus*.

Predators can be classified into herbivores, parasites, parasitoids and true predators^1^. These categories differ not only in the type of food consumed but also in the width of their trophic niche. While some herbivores, many parasites and parasitoids frequently feed only on a few host species and are often specialists, true predators typically capture a wide variety of prey. As true predators capture a number of prey items during their lifetime they are usually generalists^2^. Some species of true predators, such as snakes^3^ or spiders^4^, only capture a few prey types. These are, however, examples of facultative stenophagy or local specialisation, with exploitation of a locally abundant prey^5^. Such predators lack specialised capture adaptations^6^.

Stenophagous specialised true predators (*sensu*^6^) that capture only certain prey and possess specialised adaptations are rather rare. The trophic width of these predators is restricted yet it includes a few representatives of a family or a few species of one genus. For example, acarophagous mites catch several other mite species^7^, araneophagous spiders catch spiders from several families^8^, aphidophagous coccinellids catch aphids of a single genus^9^, lepidopterophagous bolas spiders capture male moths of a few species belonging to distinct families^10^, and myrmecophagous spiders feed on several species of a single subfamily^11^. A theoretical monophagous true predator, one in which all individuals are adapted to exclusively exploit a single prey species throughout their life and ignore even prey closely related to the focal species, has not been discovered yet.

Spiders are the largest (most diversified) order of terrestrial true predators^12^. Most species are generalist predators, some are oligophagous, while a few are stenophagous^13^. Stenophagous specialised spiders have been found to catch other spiders, woodlice, dipterans, lepidopterans, ants, and termites^6^. Although termites are locally abundant and highly palatable, they are only exploited by a few termite-eating spiders: Salticidae^14^, Theridiidae^15^, Zodariidae^16^ and Ammoxenidae^17^. This is probably because the majority of termites live underground or in wood and have highly unpredictable and brief activity on the surface, restricting their accessibility to spiders^18^.

All representatives of the family Ammoxenidae appear to be adapted to catch termites^19^. Some Ammoxenidae have been reported to feed only on the harvester termite, *Hodotermes mossambicus* (Hagen) (Isoptera: Hodotermitidae), which is the only species of the genus occurring in southern and eastern Africa^20^. *Hodotermes mossambicus* inhabits subterranean nests and forage on grass upon the surface during short activity periods^21^,^22^. Anecdotal observations suggest that some *Ammoxenus* species may be specialists of this termite species^17^,^21^,^22^,^23^,^24^,^25^.

Rigorous analysis of the trophic niche of a predator can be based on direct observations of prey consumption or on the morphological analysis of prey remnants. The latter can be successfully used only in sedentary species, such as web-building spiders, that store remnants in their web^26^. In cursorial species it is difficult to investigate the trophic niche using any of these methods, as they feed cryptically (e.g. during the night or under vegetation) and do not store prey remnants. Molecular approaches provide useful tools for determining the dietary breadth of such predators. Prey DNA sequences in the gut content of predators have been successfully analysed in many studies^27^,^28^,^29^,^30^. Next generation sequencing (NGS), in particular, allows reliable, rapid and simultaneous amplification, and subsequent identification, of thousands of prey DNA sequences from the guts of many individual predators separately^31^,^32^. It is necessary to target short prey amplicons (to ensure they survived digestion in the predator^27^,^33^), amplify part of the barcoding region of the cytochrome c oxidase I gene (to maximise the chances of identification from databases such as GenBank and BOLD, the Barcoding of Life Database), and, where possible, design primers that will amplify the DNA of a range of prey but not that of the predator^28^. Such primers must amplify sequences that are variable enough to distinguish taxa to the highest level possible^34^,^35^.

Here we focused on *Ammoxenus amphalodes* Dippenaar & Meyer, in which preliminary observations suggest that it may only capture *H. mossambicus* harvester termites^21^. We tested the hypothesis that *A. amphalodes* are monophagous, feeding on a single termite species. *Ammoxenus amphalodes* is a common, widespread species that is endemic to South Africa. It mainly inhabits open plains in grassland and savannah habitats, and usually lives in sandy soils near to *H. mossambicus* nests. We investigated the natural prey of these spiders by means of NGS. Then we investigated their willingness to accept alternative sympatrically-occurring prey types under standardised laboratory conditions. Finally, we observed their predatory behaviour and prey capture efficiency to reveal their behavioural and venomic adaptations.

## Results

### Trophic niche

The primers (AMF1 and AMR1) successfully amplified DNA from all potential prey taxa. The new primers were specific enough to amplify DNA from all the potential prey, but not that of *A. amphalodes*. Altogether, prey DNA fragments were successfully amplified from 46 females, 34 males and seven juveniles. In four females and three males (7.5%) no fragments were amplified. Ion Torrent sequencing gave us, after filtering, 1,755,965 sequences (70% of all reads). 1,371,138 sequences were used in further analyses after removing rare haplotypes and sequences with indels changing the reading frame. The number of sequences per spider varied from 5 to 101,242. Generally, fewer sequences were obtained from samples that were extracted from agarose gels (due to presence of very strong dimers during PCRs) than from samples that were purified without cutting from the gels. MOTU analysis showed 16 variants, ranging from 1 to 4 “MOTUs” (groups of haplotypes differing by 4 bp) per spider. Such variation was probably caused by sequencing errors; all but one “MOTUs” contained only 2–4 sequences.

The great majority of sequences were assigned to *H. mossambicus* (99.8% of valid sequences) when compared to GenBank and BOLD databases. Almost all those sequences were similar to *H. mossambicus* reference sequences above 99%. *Odontotermes* species differed from *H. mossambicus* only by 4 bases in the amplified fragment. When searching for the specific mutations, no base combinations corresponding to the *Odontotermes* sp. were found. Only 32 valid sequences were less than 99% similar to *H. mossambicus* (according to BOLD database criterion) but were not assigned to any other species. Two identical sequences were not assigned to any arthropod taxon with a match higher than 95%.

**Figure 1:**
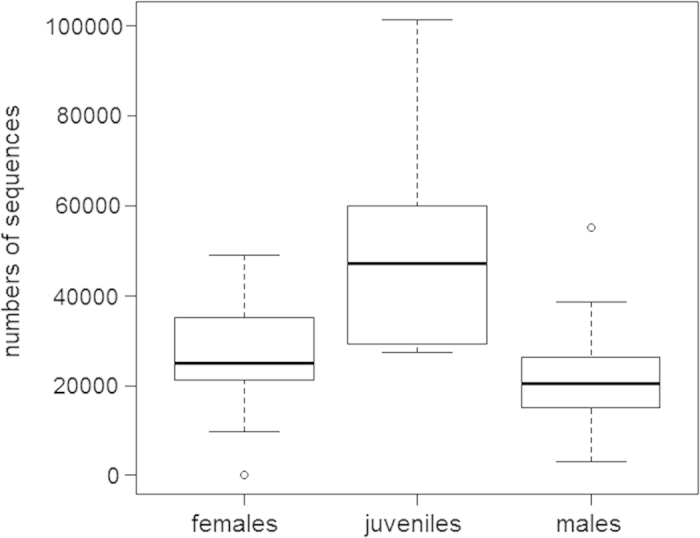
Comparison of the mean numbers of *Hodotermes mossambicus* termite sequences found in the guts of females (N = 17), males (N = 23) and juveniles (N = 7) of *Ammoxenus amphalodes* spiders.

There was a significant difference in the number of sequences (from DNA not cut from gels, N = 46) obtained from females, males and juveniles (GLM-p: F_2,43_ = 32204, P < 0.0001) (Fig. 1). The highest number of termite sequences was found in juveniles.

Seven arthropod orders were found as potential prey of *A. amphalodes* spiders (Table 1) in the two field plots. *Hodotermes mossambicus* was not a frequent prey item on the soil surface, where *A. amphalodes* foraged. The majority of potential prey were ants (91%, N = 701). Comparison of the potential and actual prey showed a significant difference (χ^2^_9_ = 459.6, P < 0.0001). Smith’s index of trophic niche is 0.07 indicating very high level of stenophagy.

### Prey acceptance

All 32 female spiders accepted *H. mossambicus* as prey but none even attempted to catch other termite species or any other prey. They did not even emerge from the soil to make contact with the alternative prey offered to them.

### Predatory behaviour

**Figure 2:**
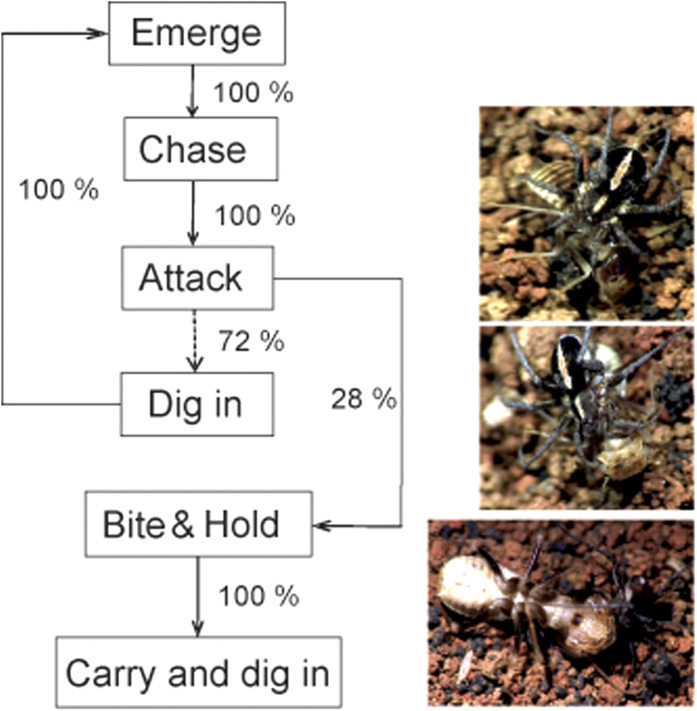
Flow diagram of hunting sequence of *Ammoxenus amphalodes* spiders (N = 32) when hunting *Hodotermes mossambicus* termites. Percentages signify the proportion of individuals transferring from previous step to the next one. Pictures show particular behaviours (attack, holding and carrying, burying beneath prey). Photographs made by Stano Pekár.

The hunting sequence was composed of the following events: emerge (the spider emerged from the sand), chase (chasing the moving termite), attack (the spider aimed to attack the termite), bite (the spider bit the termite and held it), and dig in (the spider dragged the immobilized termite and dug with it into the soil) (see Supporting information). The flow diagram (Fig. 2) shows that 72% (N = 32) of spiders needed more than one attempt to catch the termite, repeatedly digging themselves in and out of the soil and making sequential attacks. The remaining individuals (28%) successfully captured the termite at the first attempt. A successful bite was inflicted behind the termite’s head on the lateral side of the thorax. On average (1.96 ± 0.22 (SE)), spiders captured the termite on the second attempt. The termite struggled hard after being bitten, but the spider held it firmly while lying on its back. Then the spider dug itself into the soil together with the immobilized termite and fed on the termite while hidden in the soil.

**Figure 3:**
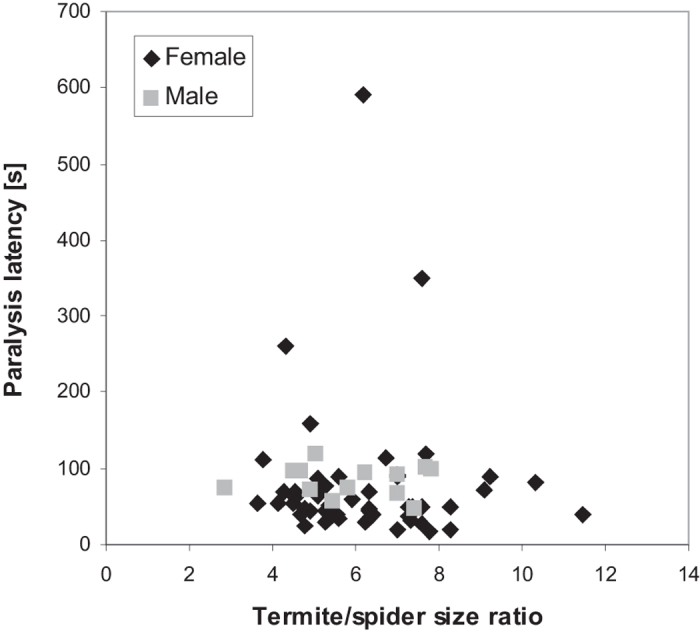
Relationship between paralysis latency after final bite and termite/spider body size ratio in female and male *Ammoxenus* spiders (N = 40). The GLM revealed no relationship (GLM-g: χ^2^_1_ = 0.05, P = 0.84).

It took a mean of 82 s (SE = 10.5) to immobilize a termite completely. The paralysis latency did not differ between male and female spiders (N = 40; GLM-g: χ^2^_1_ = 0.05, P = 0.83), and there was no relationship between termite/spider size ratio and the paralysis latency (N = 40; GLM-g: χ^2^_1_ = 0.05, P = 0.84, Fig. 3).

## Discussion

This paper appears to be the first where invertebrate predators have been individually screened using NGS. It is also one of very few papers to use NGS to analyze the diet of an invertebrate species^36^. The combination of prey DNA analysis with behavioural experiments allowed us to obtain more reliable data on a species’ diet than any single approach. The results of the DNA analyses were in close concordance with data from the behavioural experiments.

Unlike all other papers^29^,^31^,^32^,^35^,^36^,^37^,^38^ using NGS to analyse diet to date only one prey species was detected and identified (99.8% of valid sequences). Although different “MOTUs” were isolated, all had their closest match to one termite species, *H. mossambicus*. Although some MOTU could have belonged to different termite haplotypes, or possibly nuclear copies of the mtDNA sequences within the nuclear genome were present, we think it more likely that most of the differences were caused by sequencing errors. Evidence for this comes from the very low sequence numbers (<4) of different MOTUs found within individual spider predators. Several factors have quantitative affects on DNA amplification during gut content analysis, particularly primer efficiency, DNA copies number per cell in different prey species, the effect of temperature and predator activity on DNA degradation and differences in the digestibility of different prey^29^,^39^ (e.g. soft-bodies vs. chitinous invertebrate prey). Given that (1) we used the same primers throughout, (2) there was only one prey species, and (3) all spiders and termites were living under the same microclimatic conditions (temperature), we can make an approximate comparison of the relative quantities of ingested prey. The highest number of sequences per individual was found in juvenile spiders. This is likely because juveniles may catch prey more often than adults due to their investment in ontogenetic development^40^,^41^.

Our results confirm that *A. amphalodes* is a monophagous termite-eater. However, we expected that it would catch and exploit several termite species. Although sequences for *H. mossambicus* and *Odontotermes* sp. collected at the same site were so similar, when compared to the databases no sequence was assigned to any *Odontotermes* sp. (among 34 sequences of the barcoding region available in the BOLD database), not even with a lower percentage similarity. Therefore, the likelihood that the sequences were incorrectly assigned to *H. mossambicus* is very low. The two termite species differ considerably in their ecology: *H. mossambicus* forages upon the soil surface and is thus available to *A. amphalodes*, whereas *Odontotermes* sp. inhabits wood and is thus inaccessible. Furthermore, in the laboratory *A. amphalodes* spiders did not accept other termite species. There are two other harvester termite species occurring in South Africa, *Trinervitermes trinervoides* and *Microhodotermes viator* (Latreille 1804), which have been reported to be prey of other *Ammoxenus* species^19^,^21^,^24^. However, we have not found any sequence that would correspond to either of those two species. *Microhodotermes viator* does not occur in the Free State Province where this study was conducted^20^. Thus, all our evidence shows that the population of *A. amphalodes* at our study site is a monophagous termite-eating true predator.

If *A. amphalodes* was a facultative stenophage then it would accept alternative prey when available, under laboratory conditions. Yet, our results show that not only were different prey types refused, but other sympatrically-occurring termite species were rejected. The experiments were conducted with experienced adult spiders, which might have developed a preference for *H. mossambicus* during their ontogenetic development. A previous study showed that even freshly hatched spiderlings capture *H. mossambicus*^25^, supporting suggestions for an obligate preference.

Previous continuous pitfall-trap sampling over two years revealed that both *A. amphalodes* and *H. mossambicus* are active throughout the year. However, *A. amphalodes* has two seasonal peaks—it is a bivoltine species with one reproductive period in September and the other in March. This coincides with seasonal activity of *H. mossambicus* termites but not with other sympatrically occurring termite species, namely *T. trinervoides* (Haddad, unpublished). In some places *A. amphalodes* was even observed to hide in the mounds of the latter termite species but results of our study, both field and laboratory, show that *A. amphalodes* does not feed on it. Additionally, this study found that there is a synchronised spatial co-occurrence of *A. amphalodes* and *H. mossambicus*.

In a monophagous predator all populations and all developmental stages would have to catch a single prey species. Our data were obtained from a single *A. amphalodes* population occurring in the central South Africa. Previous work on the trophic ecology of *A. amphalodes* conducted in the north-eastern part of South Africa provided evidence that this species fed exclusively on *H. mossambicus* there as well^17^,^21^,^25^. This supports our view that not only is the population we studied monophagous, but that the species as a whole is a monophagous termite-eater. Furthermore, the distributional overlap of *A. amphalodes* and *H. mossambicus* supports this view^20^,^42^.

Specialist predators must be adapted to deal with extreme predator-prey size differences, with tiny juveniles capable of capturing prey as large as those attacked by adults. Prey specialists seem to be adapted to catch extremely large prey. For example, ant-eating *Zodarion cyrenaicum* Denis that occurs in the Negev Desert captured mainly large *Messor arenarius* Fabricius ants^43^. Surprisingly, even the first instar juveniles captured this ant species, even though the ants are gigantic when compared to the body size of the spiders^44^. All ontogenetic stages of *A. amphalodes* were found to catch *H. mossambicus*^21^,^25^. The body size of workers of this termite species is sexually dimorphic^45^, so it might be possible that tiny spiderlings select the smaller female morphs while adult spiders select the larger male morphs. A similar trend has been observed in ant-eating *Zodarion* spiders^44^.

Although several ant-eating spiders of the genus *Zodarion* appear to be monophagous, as they only captured one prey species in the wild^44^, in the laboratory they accepted other ant species, though the capture was less efficient. In *A. amphalodes*, other termites did not even elicit an attack. The difference stems from the fact that prey selection in specialists can take place at various levels during the predation sequence: encounter—detection—recognition—immobilisation—capture—processing^46^,^47^. At each step of the predation sequence, specific adaptations may function as filters that exclude some of the available prey types, so that fewer prey types are left at the next step. A high level of specificity will correspond to the filtering at earlier stages in the predatory sequence. Specifically, in *A. amphalodes* alternative prey did not even elicit the hunting behaviour, suggesting that the filter is at the level prior to direct encounter, possibly through the detection of vibrations of foraging termites in the substrate^24^. This again points to monophagy of *A. amphalodes*.

It is not known which cues produced by *H. mossambicus* are used to elicit the predatory behaviour in *A. amphalodes*. The cues must be very specific and different from those produced by sympatrically occurring alternative prey. Alarm, trail or sex pheromones produced by prey have been reported for prey-specialists^48^,^49^, but these are used at a greater distance. During inactivity, *Ammoxenus* spiders hide singly in a silk cell buried in soil mounds around the entrance to the termite nest^17^. As *Hodotermes* termites begin their foraging activity their movement upon the soil surface may produce vibratory cues that subsequently elicit the predatory behaviour of *A. amphalodes*^24^.

*Ammoxenus amphalodes* seem to possess very effective venom to paralyse large individuals of *H. mossambicus*. The prey was paralysed within approx. 1 min and the paralysis efficiency was independent of predator-prey body size ratio, even for extreme ratios, when the termite was ten times larger than the spider prosoma. A paralysis latency of 30–35 s was reported for adult *A. pentheri* feeding on *H. mossambicus*^25^, while 2nd and 3rd instars took approximately 1 min to paralyze prey. In a study on paralysis efficiency of myrmecophagous spider specialists we found that the efficiency decreases with an increasing body size ratio^44^. This is likely because larger prey requires a larger volume of venom to take its effect. Absence of a relationship here suggests that either *A. amphalodes* spiders adjusted the amount of injected venom perfectly according to the prey size, or they possess very effective venom compounds. A similarly high venom efficacy has been found in ant-eating *Zodarion* spiders^44^.

*Ammoxenus amphalodes* seems to consume one termite per week, which amounts to dozens of individuals during their life cycle. The termites have, however, unpredictable foraging activity, often staying inactive for several days at a time. How does the spider deal with their absence? The silken retreats of *A. amphalodes* containing up to four immobile *H. mossambicus* termites were observed^22^. Even more surprisingly, they were not dead, but only paralyzed. The authors suggested that they could serve as food storage and be used during periods when termites are inactive.

We conclude that both the DNA analyses and laboratory experiments support the hypothesis that *A. amphalodes* is a specialized predator of a single termite species, *H. mossambicus*. The evidence suggest that this is the first case of a monophagous true predator, although we cannot entirely exclude the possibility that in other sites in Africa, where different sympatric termite species are found, they may take other species too. As in specialised herbivores, parasites and parasitoids, monophagy in true predators seems to evolve when the prey is considerably larger than the predator. Other ammoxenid species may show similar trophic ecology. Indeed, there is anecdotal evidence that *A. pentheri* Simon is a specialised predator of *H. mossambicus*^23^, whereas *Rastellus sabulosus* Platnick & Griffin feeds only on *Psammotermes allocerus* Silvestri (Isoptera: Rhinotermitidae)^50^.

## Methods

### Potential prey

Fieldwork was conducted in a grassland at the Amanzi Private Game Reserve near Brandfort (S 28° 35’ 53″ E 26° 25’ 04″), South Africa in March 2013. *Ammoxenus* spiders are rare free-living soil-dwellers hiding in the termite mounds^25^, thus are difficult to catch. Observations from previous years revealed that in March there is a seasonal peak of adult occurrence (and reproduction) of *A. amphalodes* (Haddad, unpublished). At this time females were expected to be maximising prey capture, therefore the chances to observe prey capture should be high. *Ammoxenus amphalodes* were found in a strip of short grass and bare soil, about 20 m wide and 200 m long, between two fields of cultivated Pangola grass (*Digitaria eriantha*). Previous observations revealed that *A. amphalodes* are active during the day (Haddad, pers. observ.). To investigate the potential prey of *A. amphalodes*, two square plots, each 10 × 10 m, were marked in the morning when the spiders were active. These plots were at least 20 m from each other. Arthropods occurring in the plots with body size between 3–15 mm were counted and recorded by visual census of the entire area which lasted for a period of 2 hours. The survey provided not complete but basic idea of the potential prey. Most arthropods were determined to order level, but ants were determined to genus level. Three representatives of each arthropod taxon found were stored in 100% ethanol. These included *Hippodamia* sp. (Coleoptera: Coccinellidae), *Zonocerus* sp. (Orthoptera: Acrididae), *Hodotermes mossambicus*, *Odontotermes* sp. (Isoptera: Termitidae: Termitinae), *Trinervitermes trinervoides* (Sjöstedt) (Isoptera: Termitidae: Nasutitermitinae), *Ligariella* sp. (Mantodea: Mantidae), *Pheidole* sp. (Hymenoptera: Formicidae: Myrmicinae), Lygaeidae (Hemiptera: Heteroptera), *Nysius* sp. (Hemiptera: Orsilidae), and *Thanatus vulgaris* Simon (Araneae: Philodromidae). DNA from these specimens was later used as non-target species for sequence alignment during primer design and for primer testing (see below).

### Actual prey

To investigate the actual prey of *A. amphalodes*, spiders were hand-collected with tubes in the strip of short grass over the course of a few days, at different times, at Amanzi Private Game Reserve. A total of 130 spiders were collected and immediately placed in ethanol. Six *A. amphalodes* individuals were starved to death which occurred after at least one month. The DNA of these individuals and DNA from potential prey was extracted using the salt precipitation method.

As the DNA in the gut of spiders is progressively degrading due to digestion^51^, it was necessary to use primers that amplified fragments <300 bp^27^,^52^ which are still variable enough to distinguish between taxa^34^,^35^. In the starved spiders and the potential prey, the barcoding region of the COI gene was first amplified using the LCO and HCO primers^53^ and Go Taq G2 Flexi DNA Polymerase (Promega) under the following conditions: initial denaturation at 94 °C for 5 min; 35 cycles of 94 °C for 30 s, 48 °C for 30 s as an annealing temperature, 72 °C for 1 min; and a final extension at 72 °C for 7 min. The reaction mixture total volume of 20 μL consisted of 8.3 μL nuclease-free water, 4 μL of 5x Green GoTaq buffer, 2.5 μL of 25 mM MgCl_2_, 1 μL of 10 mM dNTP’s, 1 μL of 10 μM forward and 1 μL of reverse primer, 0.2 μL of GoTaq G2 Flexi DNA polymerase (5 u/μL) and 2 μL of DNA. PCR products were detected by electrophoresis in 2% SafeView-stained agarose gels. Amplified products were sequenced on an ABI Prism 3130 Genetic Analyzer (Applied Biosystems). Sequences were aligned using Mega 5.1^54^. Additional sequences downloaded from the GenBank database, representing another African termite and spiders (accession numbers NC_018122.1, JF302834.1, JF302835.1, JF302833.1, JF302832.1, JF302836.1, JF923296.1, JX023555.1), were included in the alignment. Two primer pairs, which would amplify DNA of potential prey, but not the spiders, were designed using Amplicon.b08^55^. The primers were tested to determine PCR conditions for successful amplification of all the potential prey. The primer pair AMF1: 5′- AGCAGGAATAGTAGGAACAT-3′ and AMR1: 5′-CCWCTTTCWACTATTCTTC-3′, which amplified a 250 bp fragment, was chosen for subsequent analyses and modified with MID identifiers (10 bp tags) and Ion Torrent adaptors. We used 10 MIDs added to the forward primers and 10 different MIDs added to the reverse primers to assign prey sequences to individual predators. This gave us the capacity to separate sequences derived from the gut contents of up to 100 individual spiders^31^.

DNA was extracted from 94 spider abdomens using the DNeasy Blood & Tissue Kit (Qiagen) following the manufacturer’s protocol (purification of DNA from animal tissues) with a change in a final elution step (only 80 μL of AE buffer were used). In total, seven juveniles and 87 adults (37 males and 50 females) were screened. PCR reactions were performed using the Multiplex PCR kit (Qiagen) under the following conditions: initial denaturation at 95 °C for 15 min; 43 cycles of 94 °C for 30 s, 47.2 °C for 90 s (annealing temperature), 72 °C for 90 s; and a final extension at 72 °C for 10 minutes. Reaction mixture total volume of 20 μL consisted of 10.6 μL of Multiplex PCR Master Mix, 1.8 μL of Q-Solution, 3 μL of RNase-free water, 0.8 μL of 10 μM forward and 0.8 μL of reverse primers, and 3 μL of DNA. PCR products were detected by electrophoresis in 2% SafeView-stained agarose gels. Samples which did not form visible dimers (or only very weakly) were purified using QIAquick PCR Purification Kit (Qiagen). In cases where dimers were strong, DNA was cut from the agarose gel and extracted using the QIAquick Gel Extraction Kit (Qiagen). Both extractions and purifications were performed according to the manufacturer’s protocols. Concentration of all PCR products was assessed by comparison with the 100 bp ladder (BioLabs). Then, 5 μL of 50 μg/μL PCR products was pooled into the same sterile vial and sent for sequencing. Enrichment (emPCR) and one-directional sequencing on an Ion Torrent PGM with a 316 chip was performed at the Centre de Recerca en Agrigenòmica (Bellaterra - Barcelona, Spain).

The sequences were processed using the Galaxy platform (https://usegalaxy.org/)^56^ and BioEdit 7.2.5^57^. Reads were split according to their MIDs (with two mismatches and two deletion thresholds allowed), resulting in files corresponding to individual spiders. Sequences were filtered according to their length (<200 bp) to remove dimers or too short reads. The sequences were collapsed and rare haplotypes (containing <2 identical sequences) were removed as well as sequences with stop codons and indels changing the reading frame to eliminate sequencing errors. The remaining haplotypes were clustered into MOTUs (= molecular operational taxonomic units) using jMOTU 4.1^58^ with a 4-bp cut-off (corresponding to 1.6% sequence divergence), following^31^,^32^,^37^. The variation among species is greater than 2% (or even more than 4% in some taxa) when using COI^34^. We decided to use a lower threshold because *H. mossambicus* and *Odontotermes* sp. differed by only 4 bp in the targeted COI region. Each MOTU was compared to the GenBank database (http://blast.ncbi.nlm.nih.gov/Blast.cgi) using megablast, the BOLD database (http://www.boldsystems.org/) and also to sequences obtained from the potential prey specimens. In BOLD, sequences are assigned to species level when their similarity is higher than 99%. Some sequences (0.2%) did not match with any known sequence. One of them (0.06% of all analyzed sequences) did not appear to be a valid COI sequence; another two (0.13%), that looked valid, could not be assigned to any known taxonomic group and were probably caused by sequencing errors.

### Predatory behaviour

Forty adult *A. amphalodes* were collected at Amanzi in order to investigate the prey-capture behaviour of the spider. After transfer to the laboratory, adult spiders of both sexes were placed separately into an arena consisting of 250 ml plastic bottles (8 cm in diameter, 15 cm tall) containing a 3 cm layer of sand in the bottom. They were left for three days to settle down at room temperature (~25 °C) and a natural LD (12:12) regime, during which time they were starved. Spiders usually dug themselves into the sand immediately after being released into the arena. *Hodotermes mossambicus* termites from one nest were collected in a suburban grassland in Langenhoven Park, Bloemfontein and kept together in plastic containers (40 cm in diameter) filled with soil. Trials began when a termite was introduced to the arena occupied by a spider. The hunting sequence was observed and recorded using handycam Canon Legria HF G10. If the spider did not start hunting within one hour, the prey was removed. We measured the paralysis latency as the time between successful attack and termite immobilization. After the trial the whole body length of termites and prosoma length of the spider were measured with a Nikon SMZ800 stereomicroscope.

### Prey acceptance

The acceptance of different prey by *A. amphalodes* spiders was investigated in the lab. We used only adult females (N = 32) collected in the Amanzi with a mean body size of 5 mm as these are generally more voracious than males. The following prey that occurred syntopically with the spiders were used: termites (*H. mossambicus* workers, 9 mm body length, *T. trinervoides* workers, 4 mm, *Reticulitermes* sp., 3.5 mm), ants (*Messor* sp. workers, 7 mm; *Anoplolepis custodiens* (F. Smith) workers, 5 mm), and Tenebrionidae beetles (*Zophosis boei* Solier, 8 mm). Spiders were placed separately into the arenas (as above) and starved for two days. The prey were collected one day prior to running the trials. The prey was released into the container occupied by the spider and the result was recorded. In each trial we recorded whether the spider attacked and consumed the prey. When the spider did not attack the prey after an hour, the prey was removed from the dish and was replaced with a different prey. Each spider was offered all prey types in a randomised order. When the spider captured the prey the next prey was offered two days later. The design used was a complete block. To control for motivation to hunt the prey, *H. mossambicus* termites were offered to individuals that refused alternative prey (as a control). Only individuals that subsequently captured *H. mossambicus* termites were included in the analysis.

All analyses were performed within an R environment^59^. The composition of potential prey (i.e. relative frequency of each prey species in the field plots) was compared with the composition of the actual prey (i.e. relative frequency of spider with DNA of each prey species found in the gut) using the χ^2^ goodness of fit test. Smith’s index^60^ was used to estimate the width of the trophic niche. To compare the paralysis latency, and the relationship between body size ratio and paralysis latency, we used General Linear Model (GLM) with Gamma error structure (GLM-g). GLM with Poisson error structure (GLM-p) was used to compare the number of sequences among juveniles, females and males.

## Additional Information

**Accession codes**: The sequences used for primer design are available via GenBank (Accession numbers: KP748184-KP748191).

**How to cite this article**: Petráková, L. *et al*. Discovery of a monophagous true predator, a specialist termite-eating spider (Araneae: Ammoxenidae). *Sci. Rep*. **5**, 14013; doi: 10.1038/srep14013 (2015).

## Supplementary Material

Supplementary Information

Supplementary Movie S1

## Figures and Tables

**Table 1:** Comparison of relative frequency of potential and actual prey of *Ammoxenus amphalodes* spiders.

Prey	Potential	Actual
*H. mossambicus* (Isoptera)	0.079	1.000
*Odontotermes* sp. (Isoptera)	0.001	0.000
*Anoplolepis* sp. (Formicidae)	0.633	0.000
*Monomorium* sp. (Formicidae)	0.152	0.000
*Pheidole* sp. (Formicidae)	0.026	0.000
*Crematogaster* sp. (Formicidae)	0.021	0.000
Orthoptera	0.062	0.000
Heteroptera	0.007	0.000
Coleoptera	0.011	0.000
Mantodea	0.003	0.000
Araneae	0.005	0.000
Total	758	87

Potential prey is the proportion of prey individuals found at the study site. Actual prey is the proportion of *Ammoxenus* individuals with corresponding prey sequences in their gut.
