# Supplementary material for: Discovery of a monophagous true predator, a specialist termite-eating spider (Araneae: Ammoxenidae)
Source: Sci Rep. 2015 Sep 11;5:14013. doi: 10.1038/srep14013 (PMC4566138; doi:10.1038/srep14013)
Supplement: Supplementary Information [file srep14013-s1.doc]

**Title:** Discovery of a monophagous true predator, a specialist termite-eating spider (Araneae: Ammoxenidae)

**Authors**: Lenka Petráková^1^, Eva Líznarová^1^, Stano Pekár^1*^, Charles R. Haddad^2^ , Lenka Sentenská^1^ & William O. C. Symondson^3^

**Supporting information.** Video of prey capture of *Hodotermes mossambicus* by *Ammoxenus amphalodes*. *Ammoxenus* runs very fast upon the soil surface near foraging *Hodotermes* termites. *Ammoxenus* performs a few unsuccessful attacks, finally biting behind the head capsule, flipping over, and burying itself while feeding on the termite.
